# Measuring the response to visually presented faces in the human lateral prefrontal cortex

**DOI:** 10.1093/texcom/tgac036

**Published:** 2022-08-18

**Authors:** Lara Nikel, Magdalena W Sliwinska, Emel Kucuk, Leslie G Ungerleider, David Pitcher

**Affiliations:** Department of Psychology, University of York, Heslington, York YO10 5DD, UK; School of Psychology, Liverpool John Moores University, Liverpool L3 3AF, UK; Department of Psychology, University of York, Heslington, York YO10 5DD, UK; Section on Neurocircuitry, Laboratory of Brain and Cognition, National Institute of Mental Health, Bethesda, MD, 20892, USA; Department of Psychology, University of York, Heslington, York YO10 5DD, UK

**Keywords:** dynamic face processing, fusiform face area (FFA), occipital face area (OFA), superior temporal sulcus (STS), visual field mapping

## Abstract

Neuroimaging studies identify multiple face-selective areas in the human brain. In the current study, we compared the functional response of the face area in the lateral prefrontal cortex to that of other face-selective areas. In Experiment 1, participants (*n* = 32) were scanned viewing videos containing faces, bodies, scenes, objects, and scrambled objects. We identified a face-selective area in the right inferior frontal gyrus (rIFG). In Experiment 2, participants (*n* = 24) viewed the same videos or static images. Results showed that the rIFG, right posterior superior temporal sulcus (rpSTS), and right occipital face area (rOFA) exhibited a greater response to moving than static faces. In Experiment 3, participants (*n* = 18) viewed face videos in the contralateral and ipsilateral visual fields. Results showed that the rIFG and rpSTS showed no visual field bias, while the rOFA and right fusiform face area (rFFA) showed a contralateral bias. These experiments suggest two conclusions; firstly, in all three experiments, the face area in the IFG was not as reliably identified as face areas in the occipitotemporal cortex. Secondly, the similarity of the response profiles in the IFG and pSTS suggests the areas may perform similar cognitive functions, a conclusion consistent with prior neuroanatomical and functional connectivity evidence.

## Introduction

Faces are rich sources of social information that convey someone’s identity, attentional focus, and emotional state. Humans process this wealth of socially relevant information in a network of face-selective areas distributed across the brain (Haxby et al. 2000; Calder and Young 2005; Pitcher et al. 2011b). Three of the most heavily studied face-selective areas are in the occipitotemporal cortex and are thought to perform different cognitive functions. The fusiform face area (FFA) preferentially processes facial identity (Grill-Spector et al. 2004; Rotshtein et al. 2005; Parvizi et al. 2012; Rezlescu et al. 2012), the posterior superior temporal sulcus (pSTS) preferentially processes facial expressions (Winston et al. 2004; Pitcher et al. 2014) and the occipital face area (OFA) processes the component parts of the face (e.g. eyes and mouth) (Gauthier et al. 2000; Rossion et al. 2003; Pitcher et al. 2007). Beyond these core face-selective areas in visual cortex, there is an extended network of additional face processing areas (Haxby et al. 2000; Calder and Young 2005). One area identified in neural models of face processing is in the lateral prefrontal cortex (Chan 2013). Studies of both humans and non-human primates report face-selective neural activity in the lateral prefrontal cortex (Haxby et al. 1995; Haxby et al. 1996; Scalaidhe et al. 1997; Ishai et al. 2002; Tsao et al. 2008; Chan and Downing 2011; Shepherd and Freiwald 2018) but how the lateral prefrontal cortex interacts with face-selective areas in the occipitotemporal cortex remains unclear. In the current study, we compared the neural response to faces in the lateral prefrontal cortex with that observed in the more commonly studied face-selective areas in the occipitotemporal cortex.

Our prior knowledge and experience of the world shapes how we perceive incoming sensory input. The lateral prefrontal cortex is implicated in several neural processes that support these processes including cognitive control (MacDonald et al. 2000), working memory (Curtis and D'Esposito 2003), and Theory of Mind (Kalbe et al. 2010), executive function (Goldman-Rakic 1996; Goldman-Rakic 2000) and the processing of salient stimuli and object- versus spatial-based attention (Bedini and Baldauf 2021). This range of different cognitive functions is consistent with evidence demonstrating that prefrontal areas are identified in face processing studies regardless of stimulus format, emotional valence, or task demands (Ishai et al. 2005). Neuroimaging studies of face processing have also demonstrated that the lateral prefrontal cortex is involved in the top-down control of ventral temporal cortex when recognizing faces (Heekeren et al. 2004; Baldauf and Desimone 2014). In addition, the lateral prefrontal cortex has been implicated in familiar face recognition (Rapcsak et al. 1996), working memory for faces (Courtney et al. 1996, 1997), famous-face recognition (Ishai et al. 2002), processing of information from the eyes (Chan and Downing 2011), and configural processing of the component parts of faces (e.g. the eyes and mouth) (Renzi et al. 2013). Such a broad range of different face processing functions suggests that the lateral prefrontal cortex may engage with other face processing areas depending on the specific requirements of the face processing task being performed.

The recognition of facial expressions of emotion is one of the functions processed in the lateral prefrontal cortex. Connectivity between the lateral prefrontal cortex and the amygdala has been demonstrated in healthy human participants (Davies-Thompson and Andrews 2012), and this same circuit is thought to be impaired in mental illnesses such as major depressive disorder (MDD) (Heller et al. 2009). More recently, a large-scale analysis of data collected from 680 participants reported a connection between the lateral prefrontal cortex and pSTS specialized for processing the dynamic facial aspects (Wang et al. 2020). The authors segregated the established nodes of the face processing network into 3 sub-networks using structural and functional connectivity analyses. Notably, results demonstrated that the face areas in the lateral prefrontal cortex and the pSTS formed a functional network. This is consistent with studies demonstrating that the pSTS preferentially processes dynamic facial aspects (Puce et al. 1998; Fox et al. 2009; Pitcher et al. 2019) and facial expressions (Phillips et al. 1998; LaBar et al. 2003; Winston et al. 2004; Sliwinska et al. 2020b). In addition, a study that assessed damage to the arcuate fasciculus (a white matter tract that connects the lateral temporal lobe with the inferior frontal lobe) reported behavioral impairments in face based mentalizing tasks (Nakajima et al. 2018). These studies suggest that the lateral prefrontal cortex and pSTS may be nodes in a network for processing facial expressions, and particularly for processing the changes in faces that convey the emotions and intentions of other people.

The face-selective regions in the prefrontal cortex are also involved in accessing personal semantic information associated with a face. It has been suggested that they form part of a top-down sub-network, which accesses existing knowledge associated with faces and is involved in decision-making and working memory (Li et al. 2009). This is consistent with evidence showing that the inferior frontal gyrus (IFG) preferentially responds to famous faces, which, as opposed to recently learned faces, are processed beyond the stage of simple recognition to semantic identification (Leveroni et al. 2000; Ishai et al. 2005). The frontal activation may reflect long-term retrieval from a person-identity system by triggering and structuring the search for stored representations. Alternatively, the frontal regions may be part of view-independent face processing of familiar faces, as opposed to view-dependent processing of newly learned faces (Leveroni et al. 2000). This would mean that the frontal face area is involved in familiar face recognition without retrieving person-specific semantics. However, many studies support the involvement of the prefrontal areas not only in face processing but also in semantic retrieval.

Our aim was to compare the functional response of the face area in the lateral prefrontal cortex to that of other face-selective areas in the occipitotemporal cortex (namely, the OFA, FFA and pSTS). We did this by measuring the neural responses to different types of visual stimuli across the nominated face-selective regions of interest (ROIs) using functional magnetic resonance imaging (fMRI). In Experiment 1, we first established how robustly we could localize a face-selective neural response (defined using a contrast of faces greater than objects) in the lateral prefrontal cortex. We then compared the response to different categories of stimuli (faces, bodies, scenes, objects, and scrambled objects) in this area to that measured in the other face-selective areas. In Experiment 2, we measured the response to moving and static stimuli from these same visual categories across the face-selective areas. Prior studies have demonstrated that the pSTS exhibits a greater response to moving faces than static faces (Fox et al. 2009; Pitcher et al. 2019), but this same dissociation is not consistently observed in the FFA and OFA (Pilz et al. 2009; Schultz et al. 2013; Pitcher et al. 2014). Finally, in Experiment 3, we presented face videos depicting different facial expressions in the contralateral and ipsilateral visual fields. This was done to compare the visual field responses across the occipitotemporal face-selective areas with that of the face-selective area in the lateral prefrontal cortex. Prior studies have demonstrated that the contralateral visual field advantage observed in the FFA and OFA (Hemond et al. 2007; Kay et al. 2015) is absent in the STS (Pitcher et al. 2020; Sliwinska et al. 2020a; Finzi et al. 2021). We hypothesized that if the face areas in the pSTS and lateral prefrontal cortex perform similar cognitive operations (e.g. expression recognition), then the lateral prefrontal cortex would also show an equal response to faces in both visual fields (thus distinguishing it from the FFA and OFA).

## Materials and methods

### Participants

In Experiment 1, a total of 32 right-handed participants (18 females, 14 males) with normal, or corrected-to-normal, vision gave informed consent as directed by the Ethics committee at the University of York. In Experiment 2, 24 right-handed participants (17 females, 7 males) with normal, or corrected-to-normal, vision gave informed consent as directed by the Ethics committee at the University of York. In Experiment 3, 18 participants (10 females, 8 males) with normal, or corrected-to-normal, vision gave informed consent as directed by the National Institutes of Mental Health (NIMH) Institutional Review Board (IRB). The data reported in Experiment 3 were collected for a previous visual field mapping experiment (Pitcher et al. 2020) and re-analyzed for the current study.

### Stimuli

In all 3 experiments, we used 3-s movie clips of faces and objects to localize the face-selective brain areas of interest (Pitcher et al. 2011a; Pitcher et al. 2014; Sliwinska et al. 2022). In Experiments 1 and 2, participants also viewed 3-s movie clips of bodies, scenes, and scrambled objects to calculate the response profiles to different stimulus categories. There were 60 movie clips for each category in which distinct exemplars appeared multiple times. Movies of faces and bodies were filmed on a black background, and framed close-up to reveal only the faces or bodies of 7 children as they danced or played with toys or adults (who were out of frame). Fifteen different locations were used for the scene stimuli, which were mostly pastoral scenes shot from a car window while driving slowly through leafy suburbs, along with some other films taken while flying through canyons or walking through tunnels that were included for variety. Fifteen different moving objects were selected that minimized any suggestion of animacy of the object itself or of a hidden actor pushing the object (these included mobiles, windup toys, toy planes and tractors, balls rolling down sloped inclines). Scrambled objects were constructed by dividing each object movie clip into a 15 by 15 box grid and spatially rearranging the location of each of the resulting movie frames. Within each block, stimuli were randomly selected from within the entire set for that stimulus category (faces, bodies, scenes, objects, scrambled objects). This meant that the same actor, scene, or object could appear within the same block but given the number of stimuli, this did not occur regularly.

**Fig. 1:**
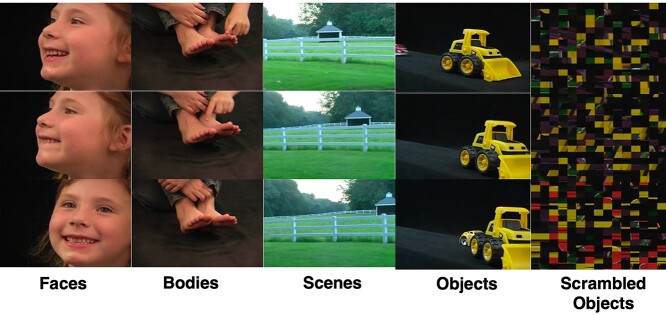
Examples of the static images taken from the 3-s movie clips depicting faces, bodies, scenes, objects, and scrambled objects. Still images taken from the beginning, middle, and end of the corresponding movie clip.

In Experiment 2, static stimuli were identical in design to the dynamic stimuli except that in place of each 3-s movie, we presented 3 different still images taken from the beginning, middle, and end of the corresponding movie clip. Each image was presented for one second with no ISI, to equate the total presentation time with the corresponding dynamic movie clip (Fig. 1).

In Experiment 3, visual field responses in face-selective regions were mapped using 2-s video clips of dynamic faces making one of 4 different facial expressions: happy, fear, disgust, and neutral air-puff. These faces were used in a previous fMRI study of face perception (van der Gaag et al. 2007). Happy expressions were recorded when actors laughed spontaneously at jokes, whereas the fearful and disgusted expressions were posed by the actors. The neutral, air-puff condition consisted of the actors blowing out their cheeks to produce movement but expressing no emotion. Both male and female actors were used. Videos were filmed against a gray background and the actors limited their head movements. Face videos were presented in the contralateral and ipsilateral visual hemifields at 5 by 5 degrees of visual angle and shown at a distance of 5 degrees from fixation to the edge of the stimulus (Pitcher et al. 2020).

### Procedure and data acquisition

#### Experiment 1—Localizing the face-selective area in the lateral prefrontal cortex

Functional runs presented movie clips from 5 different stimulus categories (faces, bodies, scenes, objects, or scrambled objects). Data were acquired over 6 blocked-design functional runs lasting 234 s each. Each functional run contained three 18-s rest blocks, at the beginning, middle, and end of the run, during which a series of 6 uniform color fields were presented for 3 s each. Participants were instructed to watch the movies but were not asked to perform any overt task.

Each run contained two sets of 5 consecutive stimulus blocks (faces, bodies, scenes, objects, or scrambled objects) sandwiched between these rest blocks, to make 2 blocks per stimulus category per run. Each block lasted 18 s and contained stimuli from one of the 5 stimulus categories. The order of stimulus category blocks in each run was palindromic (e.g. fixation, faces, objects, scenes, bodies, scrambled objects, fixation, scrambled objects, bodies, scenes, objects, faces, fixation) and was randomized across runs.

Imaging data were collected using a 3 T GE HDx Excite MRI scanner at the University of York. Functional images were acquired with an 8-channel phased array head coil (GE) and a gradient-echo EPI sequence (38 interleaved slices, repetition time (TR) = 3 s, echo time (TE) = 30 ms, flip angle = 90 degrees; voxel size 3 mm isotropic; matrix size = 128 × 128) providing whole brain coverage. Slices were aligned with the anterior to posterior commissure line. Structural images were acquired using the same head coil and a high-resolution T-1 weighted 3D fast spoilt gradient (SPGR) sequence (176 interleaved slices, repetition time (TR) = 7.8 s, echo time (TE) = 3 ms, flip angle = 20 degrees; voxel size 1 mm isotropic; matrix size = 256 × 256).

#### Experiment 2—Measuring the response to moving and static stimuli in the right IFG

Functional data were acquired over 11 blocked-design functional runs lasting 234 s each. Each functional run contained three 18-s rest blocks, at the beginning, middle, and end of the run, during which a series of 6 uniform color fields were presented for 3 s. Participants were instructed to watch the movies and static images but were not asked to perform any overt task.

Functional runs presented either movie clips (the 8 dynamic runs) or sets of static images taken from the same movies (the 4 static runs). For the dynamic runs, each 18-s block contained six 3-s movie clips from that category. For the static runs, each 18-s block contained 18 one-s still snapshots, composed of 6 triplets of snapshots taken at 1-s intervals from the same movie clip. Dynamic/static runs were run in the following order: 2 dynamic, 2 static, 2 dynamic, 2 static, 4 dynamic. The final 3 runs of the dynamic stimuli were used to define face-selective ROIs (see “Data Analysis” section).

Imaging data were acquired using a 3 T Siemens Magnetom Prisma MRI scanner (Siemens Healthcare, Erlangen, Germany) at the University of York. Functional images were acquired with a 20-channel phased array head coil and a gradient-echo EPI sequence (38 interleaved slices, repetition time (TR) = 3 s, echo time (TE) = 30 ms, flip angle =90%; voxel size 3 mm isotropic; matrix size = 128 × 128) providing whole brain coverage. Slices were aligned with the anterior to posterior commissure line. Structural images were acquired using the same head coil and a high-resolution T-1 weighted 3D fast spoilt gradient (SPGR) sequence (176 interleaved slices, repetition time (TR) = 7.8 s, echo time (TE) = 3 ms, flip angle = 20 degrees; voxel size 1 mm isotropic; matrix size = 256 × 256).

#### Experiment 3—Measuring the visual field response in the face area in the IFG

**Fig. 2:**
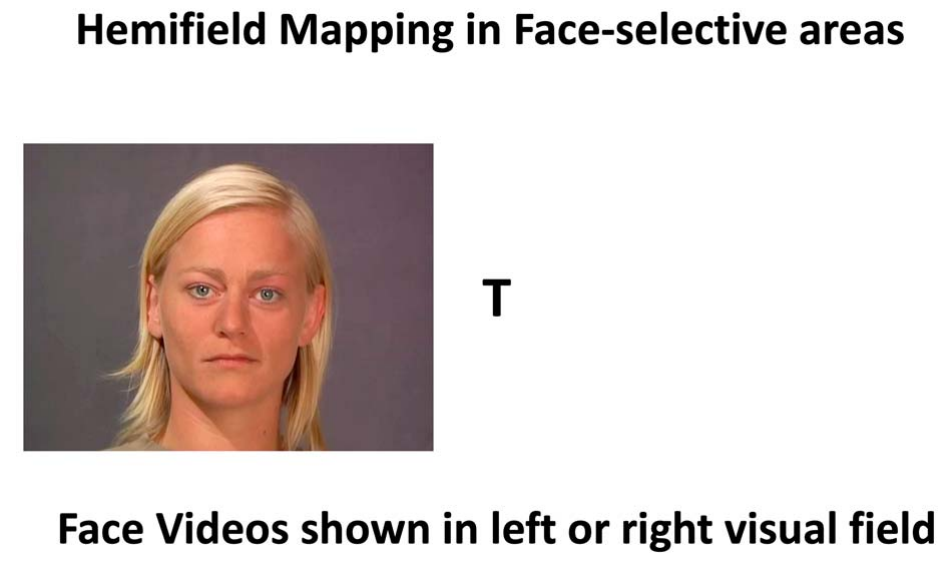
Static image taken from the hemifield visual field (VF) mapping stimulus used in Experiment 3. Actors displaying different emotions (happy, fear, disgust, neutral air-puff) were shown in the two hemifields. Participants maintained fixation by detecting the presence of either a T or an L (shown upright or inverted) at fixation (Pitcher et al. 2020).

Participants fixated the center of the screen while 2-s video clips of actors performing different facial expressions were shown in the 4 quadrants of the visual field. To ensure that participants maintained fixation, they were required to detect the presence of an upright or inverted letter (either a T or an L) at the center of the screen. Letters (0.6° in size) were presented at fixation for 250 ms in random order and in different orientations at 4 Hz (Kastner et al. 1999). Participants were instructed to respond when the target letter (either T or L) was shown; this occurred approximately 25% of the time. The target letter (T or L) was alternated and balanced across participants. We informed the participants that the target detection task was the aim of the experiment, and we discarded any runs in which the participant scored less than 75 percent correct (Fig. 2).

Visual field mapping images were acquired over 6 blocked-design functional runs lasting 408 s each. Each functional run contained sixteen 16-s blocks during which 8 videos of 8 different actors performing the same facial expression (happy, fear, disgust, and neutral air-puff) were presented in one of the two hemifields. Eight blocks were shown in each hemifield and the order in which they appeared was randomized. After the visual field mapping blocks were completed, participants completed 6 blocked-design functional runs lasting 234 s each to functionally localize the face-selective ROIs.

Imaging data were acquired using research dedicated GE 3-Tesla MR 750 scanner at the National Institutes of Health (NIH). Functional images were acquired with a 32 channel phased array head coil and a gradient-echo EPI sequence (36 interleaved slices, repetition time (TR) = 2 s, echo time (TE) = 30 ms, flip angle =77%; voxel size 3 mm isotropic; matrix size = 128 × 128) providing whole brain coverage. Slices were aligned with the anterior to posterior commissure line. In addition, a high-resolution T-1 weighted MPRAGE anatomical scan (T1-weighted FLASH, 1 × 1 × 1 mm resolution) was acquired to anatomically localize functional activations.

### Imaging analysis

Functional MRI data were analyzed using AFNI (http://afni.nimh.nih.gov/afni). Images were slice-time corrected and realigned to the third volume of the first functional run and to the corresponding anatomical scan. All data were motion corrected and any TRs in which a participant moved more than 0.3 mm in relation to the previous TR were discarded from further analysis. The volume-registered data were spatially smoothed with a 4-mm full-width-half-maximum Gaussian kernel. Signal intensity was normalized to the mean signal value within each run and multiplied by 100 so that the data represented percent signal change from the mean signal value before analysis.

**Fig. 3:**
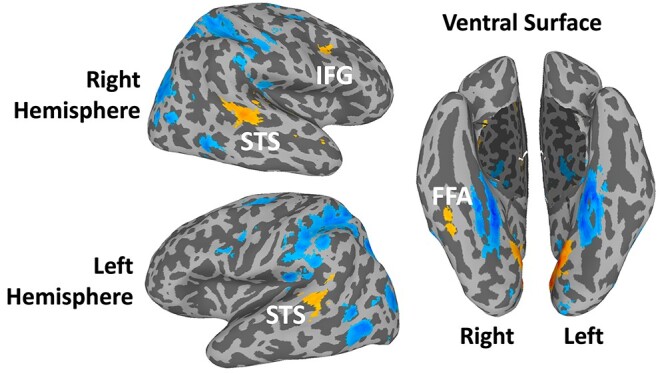
Results of a whole-brain group analysis (*n* = 32) showing a contrast of moving faces greater than moving objects activations on the lateral surfaces of an inflated brain surface (t-statistical threshold is *P* = 0.001, cluster correction of 50 voxels). Faces > objects activations are shown in orange, and objects > faces activations are shown in blue. Generated using the probabilistic maps for combining functional imaging data with cytoarchitectonic maps (Eickhoff et al. 2005).

In Experiment 1, data from all 6 runs were entered into a general linear model (GLM) by convolving the standard hemodynamic response function with the regressors of interest (faces, bodies, scenes, objects, and scrambled objects). Regressors of no interest (e.g. 6 head movement parameters obtained during volume registration and AFNI’s baseline estimates) were also included in the GLM. Data from all 32 participants were entered in a group whole brain analysis to identify the locus of the face-selective activations in the bilateral frontal cortex using a contrast of moving faces greater than moving objects (Fig. 3).

We next analyzed data for all participants individually to localize the regions of interest (ROIs). Face-selective ROIs were identified for each participant using a contrast of greater activation evoked by faces than that evoked by objects, calculating significance maps of the brain using an uncorrected statistical threshold of *P* = 0.001. In addition to the face-selective area in the prefrontal cortex, we also identified the FFA, pSTS, and OFA. Finally, we performed a split-half analysis to calculate the neural response to different stimulus categories (faces, bodies, scenes, objects, and scrambled objects) in the face-selective ROIs. Even runs (2, 4, and 6) were used to identify the face-selective areas; odd runs (1, 3, and 5) were used to calculate the neural responses. Within each functionally defined ROI, we then calculated the magnitude of response (percent signal change (PSC) from a fixation baseline) for each stimulus category. We selected all contiguous voxels for each ROI.

Data in Experiment 2 were analyzed using the same preprocessing procedures described in Experiment 1 except for the following differences. ROIs were calculated using data from 4 dynamic runs (runs 9 to 11). Face-selective ROIs were identified using a contrast of moving faces greater than moving objects using an uncorrected statistical threshold of *P* = 0.001. Within ROIs, we then calculated the magnitude of response to the dynamic and static conditions of each of the 5 stimulus categories (faces, bodies, scenes, objects, and scrambled objects), using the data collected from runs 1 to 8 in which pairs of dynamic and static runs were alternated. All the data used to calculate PSC were independent of the data used to define the ROIs.

Data in Experiment 3 were analyzed using the same preprocessing procedures described in Experiment 1 except for the following differences. Face-selective ROIs were identified using data from 6 dynamic runs (7 to 12) using a contrast of moving faces greater than moving objects using an uncorrected statistical threshold of *P* = 0.001. Within ROIs, we then calculated the magnitude of response to moving face videos presented in the contralateral and ipsilateral visual fields using the data collected from runs 1 to 6.

## Results

### Experiment 1: Localizing the face-selective area in the lateral prefrontal cortex

**Fig. 4:**
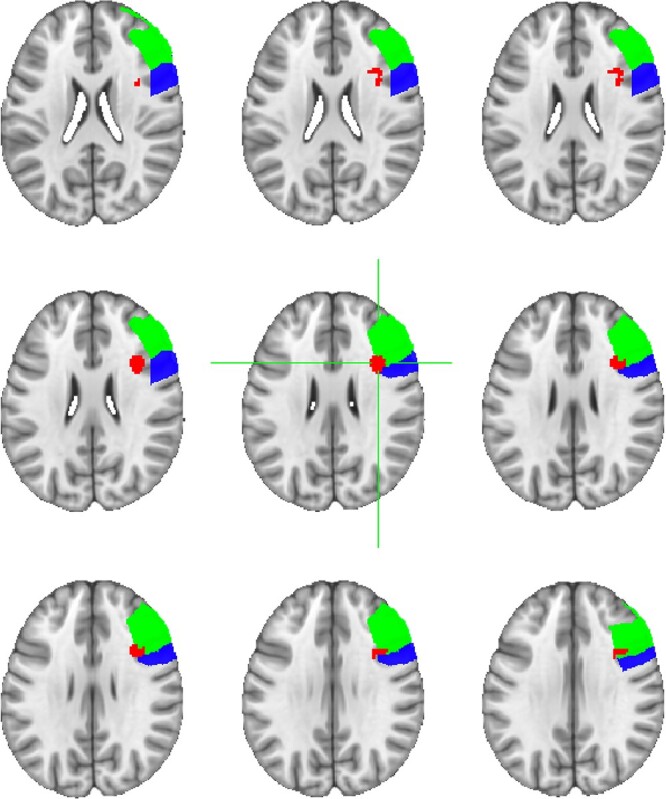
Results of a whole-brain group analysis (*n* = 32) showing a contrast of moving faces greater than moving objects activations in red (t-statistical threshold is *P* = 0.001, cluster correction of 50 voxels). The face-selective are in the right lateral prefrontal cortex is shown in red, the anatomical area of the right inferior frontal gyrus is shown in blue, and the anatomical area of the right middle frontal gyrus is shown in green. The peak face-selective voxel (MNI co-ordinates 37, 13, 28) was centered in the pars opercularis of the right inferior frontal gyrus (IFG) according to the probabilistic maps for combining functional imaging data with cytoarchitectonic maps (Eickhoff et al. 2005).

Data from all 32 participants were entered into a group whole brain ANOVA to identify the locus of the face-selective activations in the prefrontal cortex. The results of a contrast of moving faces greater than moving objects are shown in Fig. 3. Using a *t*-statistical threshold of *P* = 0.001 and a cluster correction of 50 voxels, we were able to localize a face-selective activation in the right lateral prefrontal cortex, but not in the left lateral prefrontal cortex. The face-selective activation in the right lateral prefrontal cortex (Fig. 4) was centered in the pars opercularis of the inferior frontal gyrus (MNI coordinates 37, 13, 28) according to the probabilistic maps for combining functional imaging data with cytoarchitectonic maps (Eickhoff et al. 2005). The activation was also within 1 mm of the right inferior frontal junction where face-selective activation has previously been reported by other groups (Gobbini et al. 2004; Chan and Downing 2011; Keightley et al. 2011).

To further characterize how reliable this activation was across all 32 participants, we next looked at the individual level using data collected from all 6 experimental runs. Results revealed that a face-selective area in the frontal cortex was present in 24 participants in the right hemisphere (mean MNI co-ordinates 42, 14, 32), but only 18 in the left hemisphere (mean MNI co-ordinates −38, 17, 33). By contrast, we were able to localize the right FFA (mean MNI co-ordinates 41, −52, −17), left FFA (mean MNI co-ordinates −41, −52, −17), right pSTS (mean MNI co-ordinates 53, −37, 5), and left pSTS (mean MNI co-ordinates −57, −39, 6) in 31 of 32 participants. The right OFA was present in 30 participants (mean MNI co-ordinates 40, −79, −10) and the left OFA in 25 (mean MNI co-ordinates −39, −82, −10). This greater preference for face processing in the right hemisphere is consistent with prior evidence (Young et al. 1985; Barton et al. 2002; Yovel et al. 2003; Sliwinska and Pitcher 2018).

These results demonstrate that the face-selective area in the IFG was not as reliably identified across participants as face-selective areas in the occipitotemporal cortex. To further characterize the reliability of detecting face-selective activity in the IFG, we performed additional analyses. Firstly, we measured the size of the activation across participants. The average size of the right IFG was 246 voxels (SE = 34 voxels) with a range of 41 to 583 voxels. The average size of the left IFG was 127 voxels (SE = 14 voxels) with a range of 47 to 208 voxels. Next, we were able to identify face-selective activation in the right IFG of 4 of the 8 participants who failed to show any activation at *P* = 0.001 by lowering the statistical threshold to *P* = 0.1. Finally, we performed a split-half analysis of our data for the 24 participants who exhibited face-selective activity in the right IFG. This was done to establish whether we could reliably locate the ROI in the same location across 2 datasets. We identified the peak face-selective voxel in righty IFG using data from the odd and even runs of the localizer (3 runs each). The results showed the peak voxel was in the same location in both runs for the 24 participants who had a right IFG response. The mean MNI co-ordinates for the odd runs was 42(1), 12,(1), 33(2), and was 42(1), 12,(1), 32(1) for the even runs (standard errors shown in brackets).

**Fig. 5:**
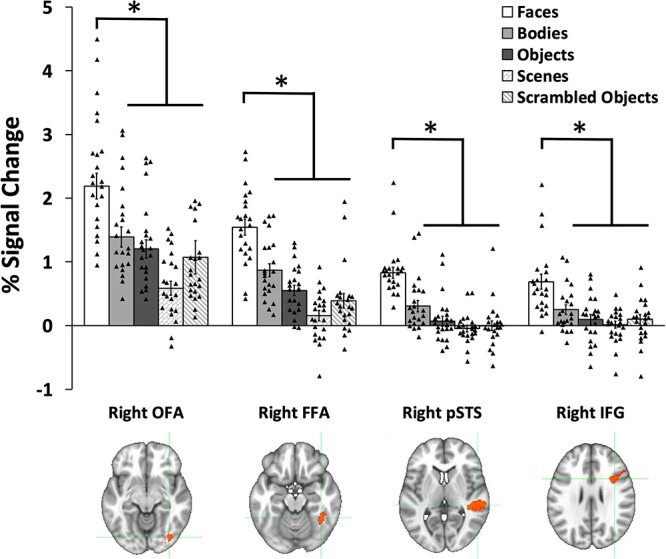
PSC data for the dynamic for 5 visual categories (faces, bodies, scenes, objects, and scrambled objects) in the rOFA, rFFA, rpSTS, and rIFG. All 4 regions showed a significantly greater response to faces than all other categories. Data shown are independent of the data used to define the ROIs. Error bars show standard errors of the mean across participants. Individual participant data are represented by the black triangles. ^*^ denotes a significant difference (*P* < 0.01) in post hoc tests.

Next, to compare the response to faces, bodies, objects, scenes, and scrambled of the face-selective area in the inferior frontal gyrus to other face-selective areas, we performed an additional split-half analysis of our data (Fig. 5). Because we were only able to localize the left IFG in 18 of the participants, we focused on the face-selective ROIs in the right hemisphere, but the responses in the left hemisphere ROIs showed the same overall pattern as those in the right hemisphere. We were able to identify the 4 ROIs of interest in 22 of the 32 participants (2 participants who had face-selective activity in the IFG did not have a right OFA).

PSC data (Fig. 5) were entered into a 2 (ROI: IFG, FFA, pSTS, OFA) by 5 (category: faces, bodies, scenes, objects, and scrambled objects) repeated measures analysis of variance (ANOVA). Results showed significant main effects of ROI (*F* (3,63) = 57, *P* < 0.001; partial η ^2^ = 0.731) and stimulus (*F* (4,84) = 72, *P* < 0.001; partial η 2 = 0.774). Stimulus and ROI also combined in a significant interaction (*F* (12,252) = 7.6, *P* < 0.001; partial η 2 = 0.265). Planned Bonferroni comparisons demonstrated that all 4 ROIs exhibited a significantly greater response to faces than to all other stimulus categories (*P* < 0.001).

### Experiment 2—Measuring the response to moving and static stimuli in the rIFG

Face-selective ROIs were identified in both hemispheres using a contrast of moving faces greater than moving objects. As in Experiment 1, we were not able to localize face-selective ROIs in all 24 participants across both hemispheres. Results revealed that a face-selective area in the frontal cortex was present in 17 participants in the right hemisphere (mean MNI co-ordinates 43, 8, 39), but only 14 in the left hemisphere (mean MNI co-ordinates −45, 2, 39). By contrast, we were able to localize the right FFA (mean MNI co-ordinates 41, −54, −17), left FFA (mean MNI co-ordinates −41, −54, −1), right pSTS (mean MNI co-ordinates 54, −43, 9), and left pSTS (mean MNI co-ordinates −55, −40, 9) in all participants. The right OFA was present in 22 participants (mean MNI co-ordinates 41, −84, −9) and the left OFA in 18 participants (mean MNI co-ordinates −40, −83, −11). We again focused our analysis on the ROIs in the right hemisphere, but the pattern in the left hemisphere ROIs was consistent.

**Fig. 6:**
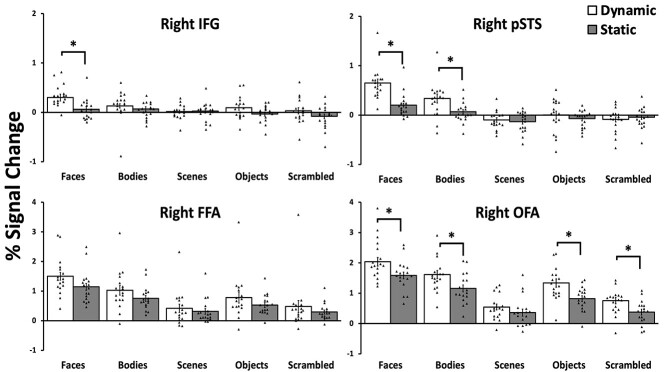
PSC data for the dynamic and static stimuli from all 5 categories (faces, bodies, scenes, objects, and scrambled objects) in the IFG, rpSTS, rFFA, and rOFA. All 4 regions showed a significantly greater response to faces than all other categories. The rIFG showed a greater response to moving faces than static faces. The rpSTS showed a greater response to moving faces than static faces and to moving bodies more than static bodies. The rOFA showed a greater response to moving more than static stimuli for 4 of the visual categories (face, bodies, objects, and scrambled objects). There was no significant difference moving and static stimuli in the rFFA. Error bars show standard errors of the mean across participants. Individual participant data are represented by the black triangles. ^*^ denotes a significant difference (*P* < 0.0001) in post hoc tests.

To establish which face-selective ROIs showed a differential response to moving and static stimuli, we analyzed the data in a 2 (motion: moving, static) by 5 (stimulus: bodies, faces, objects, scenes, scrambled objects) by 4 (ROI: FFA, OFA, pSTS, IFG) repeated-measures analysis of variance (ANOVA). We found significant main effects of motion (*F* (1,16) = 23, *P* < 0.001; partial η ^2^ = 0.587), stimulus (*F* (4,64) = 112, *P* < 0.001; partial η ^2^ = 0.875) and ROI (*F* (3,48) = 58, *P* < 0.001; partial η ^2^ = 0.784). Motion and stimulus combined in a significant interaction (*F* (4,64) = 5.7, *P* < 0.001; partial η ^2^ = 0.265). Motion and ROI combined in a significant interaction (*F* (3,48) = 3.8, *P* = 0.015; partial η ^2^ = 0.195). Stimulus and ROI combined in a significant interaction (*F* (12,192) = 25, *P* < 0.001; partial η ^2^ = 0.607). Most importantly motion, stimulus and ROI combined in a significant three-way interaction (*F* (12,192) = 2.9, *P* = 0.001; partial η ^2^ = 0.154). To further understand what factors were driving the significant effects, we then performed separate two-way ANOVAs on each face-selective ROI (Fig. 6).

### Right IFG

A 2 (motion) × 5 (stimulus) repeated-measures ANOVA showed a main effect of motion (*F* (1, 16) = 6.8, *P* = 0.019; partial η ^2^ = 0.299) with a significantly greater response to moving more than static stimuli (*P* = 0.003). There was also a main effect of stimulus (*F* (4, 64) = 9, *P* < 0.001; partial η ^2^ = 0.361) with a greater response to faces than to all other stimulus categories (*P* < 0.05). There was also a significant interaction between motion and stimulus (*F* (4, 64) = 2.5, *P* = 0.048; partial η ^2^ = 0.137). Planned Bonferroni comparisons revealed that moving faces produced a larger response than static faces (*P* < 0.001), but no other comparisons reached significance (*P* > 0. 15).

### Right pSTS

A 2 (motion) × 5 (stimulus) repeated-measures ANOVA showed a main effect of motion (*F* (1, 16) = 6.1, *P* = 0.026; partial η ^2^ = 0.290) with a significantly greater response to moving more than static stimuli (*P* = 0.026). There was also a main effect of stimulus (*F* (4, 64) = 47, *P* < 0.001; partial η ^2^ = 0.759) with a greater response to faces than to all other stimulus categories (*P* < 0.001). There was also a significant interaction between motion and stimulus (*F* (4, 64) = 13.5, *P* < 0.001; partial η ^2^ = 0.474). Planned Bonferroni comparisons revealed that moving faces produced a larger response than static faces (*P* < 0.001) and that moving bodies produced a larger response than static bodies (*P* = 0.05), but no other comparisons reached significance (*P* = 1).

### Right FFA

A 2 (motion) × 5 (stimulus) repeated-measures ANOVA showed a main effect of motion (*F* (1, 16) = 8.1, *P* = 0.012; partial η ^2^ = 0.351) with a significantly greater response to moving more than static stimuli (*P* = 0.012). There was also a main effect of stimulus (*F* (4, 64) = 61, *P* < 0.001; partial η ^2^ = 0.801) with a greater response to faces than to all other stimulus categories (*P* < 0.01). There was no significant interaction between motion and stimulus (*F* (4, 64) = 1.5, *P* = 0.2; partial η ^2^ = 0.094).

### Right OFA

A 2 (motion) × 5 (stimulus) repeated-measures ANOVA showed a main effect of motion (*F* (1, 16) = 45, *P* < 0.001; partial η ^2^ = 0.751) with a significantly greater response to moving more than static stimuli (*P* = < 0.001). There was also a main effect of stimulus (*F* (4, 64) = 53, *P* < 0.001; partial η ^2^ = 0.778) with a greater response to faces than to all other stimulus categories (*P* < 0.01). There was also a significant interaction between motion and stimulus (*F* (4, 64) = 3.6, *P* = 0.01; partial η ^2^ = 0.195). Planned Bonferroni comparisons revealed that moving faces produced a larger response than static faces (*P* < 0.001), moving objects produced a larger response than static objects (*P* < 0.001), moving bodies produced a larger response than static bodies (*P* < 0.001) and that moving scrambled objects produced a larger response than static scrambled objects (*P* = 0.01). There was no significant difference between moving and static scenes (*P* = 0.15).

### Experiment 3—fMRI mapping of faces in the two hemifields in face-selective areas

Face-selective ROIs were identified in both hemispheres using a contrast of moving faces greater than moving objects. As in Experiment 1, we were not able to localize face-selective ROIs in all 18 participants across both hemispheres. Results revealed that a face-selective area in the frontal cortex was present in 16 participants in the right hemisphere (mean MNI co-ordinates 40, 10, 32), but only 11 in the left hemisphere (mean MNI co-ordinates −43, 15, 30). We again focused our analysis on the ROIs in the right hemisphere but the pattern in the left hemisphere ROIs was consistent.

**Fig. 7:**
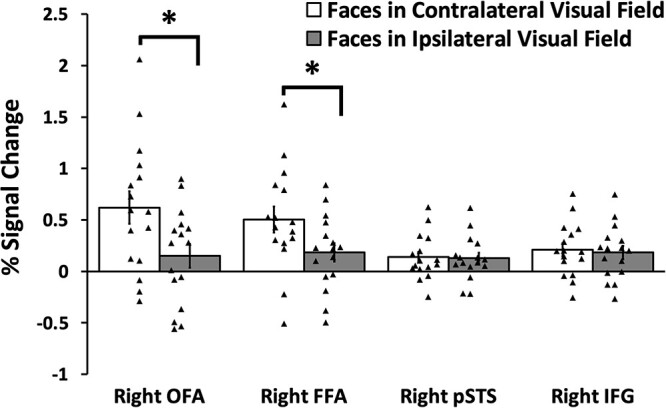
PSC for dynamic faces presented in the contralateral and ipsilateral hemifields. Results showed that the right FFA and right OFA exhibited a significantly greater response to faces in the contralateral VF than in the ipsilateral visual field. There were no visual field biases in the right pSTS or right IFG. Error bars show standard errors of the mean across participants. Individual participant data are represented by the black triangles ^*^ denotes a significant difference (*P* < 0.0001) in post hoc tests.

To establish which face-selective ROIs showed a greater response to faces in the contralateral visual field, we analyzed the data in a 2 (visual field: ipsilateral, contralateral) by 4 (ROI: FFA, OFA, pSTS, IFG) repeated-measures analysis of variance (ANOVA). We found significant main effects of visual field (*F* (1,15) = 30, *P* < 0.001; partial η ^2^ = 0.669) with a significantly greater response to faces in the contralateral more than the ipsilateral visual field (*P* < 0.001). There was no main effect of ROI (*F* (3,45) = 1.8, *P* = 0.17; partial η ^2^ = 0.105). Importantly visual field and ROI combined in a significant two-way interaction (*F* (3,45) = 31, *P* < 0.001; partial η ^2^ = 0.671). Planned Bonferroni comparisons revealed a larger response to faces in the contralateral more than ipsilateral field in the FFA (*P* < 0.001) and OFA (*P* < 0.001) but not in the pSTS (*P* = 0.5) or IFG (*P* = 0.3) (Fig. 7).

## Discussion

The aim of the current study was to measure the response to visually presented images of faces in the human lateral prefrontal cortex and to compare these responses with those recorded in the face-selective areas in the occipitotemporal cortex (FFA, pSTS, and OFA). In Experiment 1, we scanned 32 participants with fMRI while viewing short movie clips of faces, bodies, scenes, objects, and scrambled objects. Using a contrast of faces greater than objects, we identified a face-selective area centered in the pars opercularis of the right inferior frontal gyrus (IFG), a finding consistent with prior fMRI studies (Gobbini et al. 2004; Chan and Downing 2011; Keightley et al. 2011). A subsequent ROI analysis of individual participants revealed that this face-selective activation was present in only 24 of the 32 participants in the right hemisphere and in 18 participants in the left hemisphere. By contrast, the bilateral FFA and pSTS areas were present in 31 participants, the right OFA was present in 30 participants and the left OFA in 25. Even though the face area in the right IFG was less robustly identified across participants, it still exhibited the highly selective response to faces observed in the FFA, pSTS, and OFA (Fig. 5). In Experiment 2, we measured the response to moving and static stimuli across the 4 face-selective ROIs in the right hemisphere. Results demonstrated that the right IFG, right pSTS, and right OFA all exhibited a greater response to moving faces than to static faces, but the right FFA responded equally to moving and static faces (Fig. 6). Finally, in Experiment 3, we measured responses to moving faces presented in the contralateral and ipsilateral visual fields. Results demonstrated the contralateral visual field bias observed in the right FFA and right OFA was absent in the right pSTS and right IFG (Fig. 7). Taken together, the results of all 3 experiments suggest two principal conclusions. Firstly, that the face-selective area in the IFG is less robustly identified than face areas in the occipitotemporal cortex, this was observed in all 3 experiments. Secondly, that the similarity of the response patterns in the IFG and pSTS (greater response to moving faces more than static faces and no visual field bias) suggests that the two areas may perform similar cognitive functions (e.g. facial expression recognition).

The use of functional localizers in fMRI studies has been standard for over 20 (Kanwisher et al. 1997; Saxe et al. 2006), but it is known that this approach does not always identify the necessary regions of interest (ROIs) across all participants (Duncan et al. 2009; Pitcher et al. 2011a). In Experiment 1, we used 6 localizer runs to identify the face-selective ROIs, this was enough data to successfully localize the bilateral FFA, pSTS, and right OFA in all participants. However, we were only able to identify the right IFG in 24 participants and the left IFG in 18 participants. A likely explanation for this result is that we did not require subjects to perform any explicit task during the localizer runs (e.g. a one-back memory task). Such a task may not be necessary for identifying ROIs in high-level visual cortex but may be necessary for ROIs in the prefrontal cortex. The IFG has been implicated in a range of cognitive tasks including working memory, executive function, the processing of salient stimuli, and object versus spatial based attention (Goldman-Rakic 1996; Goldman-Rakic 2000; Bedini and Baldauf 2021). It is likely that future studies aiming to localize the face-selective area in the bilateral IFG should require participants to perform an explicit cognitive task in the localizer runs rather than relying on free viewing of visual stimuli as we did in the present study. This conclusion is consistent with a prior study that compared the effectiveness of localizing the IFG in a 1-back task localizer task with a free viewing localizer task (Chan and Downing 2011).

Anatomical studies in non-human primates have identified a white matter pathway, that projects from the lateral superior temporal cortex into the inferior frontal cortex.

(Kravitz et al. 2011). In humans, this pathway (the arcuate fasciculus) is more prominent than in non-human primates (Rilling et al. 2008) and is involved in a range of tasks including language (Dick and Tremblay 2012) and face processing (Nakajima et al. 2018). A large-scale study of 680 participants further characterized this pathway using structural and functional connectivity data as a specialized sub-network with the wider face processing network (Wang et al. 2020). The authors further proposed that this sub-network is specialized for processing the dynamic and changeable aspects of faces that include recognizing facial expressions and reading the intentions from a face. The results of the present study are consistent with this conclusion. In Experiment 2, we demonstrated that the right pSTS and right IFG both exhibited a greater response to moving faces than to static faces. We observed this pattern in our earlier fMRI study of moving and static faces, but we were only able to successfully localize the right IFG in 7 of 13 participants, so the result was not statistically warranted (Pitcher et al. 2011a).

The results of Experiment 2 show that all 4 face-selective areas demonstrated a greater response to moving stimuli than to static stimuli. This result is inconsistent with our prior studies that only reported a greater response to moving faces and bodies in the rpSTS and moving faces in the raSTS (Pitcher, Dilks*.* 2011a; Pitcher et al. 2019). There are methodical differences between the studies that may account for these differences. For example, in the present study, we tested more participants than in our initial study (Pitcher et al. 2011a), which is likely to have increased the statistical power. In addition, the present study used a 3 T fMRI scanner and a voxel resolution of 3 mm isotropic, while our prior study used a 7 T fMRI scanner and a voxel resolution of 1.2 mm isotropic (Pitcher et al. 2019). However, it is important to note that in all 3 studies, the difference in response to moving and static faces is greater in the rpSTS than it is the rFFA and rOFA. In addition, it should be noted that natural motion has been shown to enhance the neural response to faces in face-selective areas in other studies (Schultz and Pilz 2009; Schultz et al. 2013). This suggests that while motion can enhance the response to faces across the brain it is in the STS, and the IFG, where this difference is the greatest (Fig. 6).

Our prior visual mapping studies in humans demonstrated that the face-selective area in the pSTS lacked the contralateral visual field bias observed in the FFA and OFA (Pitcher et al. 2020; Sliwinska et al. 2020a) (see also (Finzi et al. 2021)). In the present study, we re-analyzed this earlier data and established that the face-selective area in the right IFG also exhibited no visual field bias (Fig. 7). This shared functional profile between the face-selective areas in the pSTS and IFG further suggests the two areas are connected when performing cognitive operations that involve moving faces (e.g. facial expression recognition). We have previously suggested that dynamic social interactions require tracking the movements of faces and bodies across the entire visual field, which is consistent with this finding (Pitcher and Ungerleider 2021).It is also likely the IFG is connected to our recently proposed third visual pathway for social perception (Pitcher et al. 2017; Pitcher and Ungerleider 2021), but it should be noted that the IFG is also connected to the dorsal visual pathway for action observation (Kilner 2011).

The precise role of the IFG in memory processing, its lateralization and whether it is object-specific, or domain general is unclear. Facial working memory, in which a representation of a face is maintained after it has been removed from view, activates prefrontal regions (Courtney et al. 1996, 1997). It has been proposed that right frontal activity may be associated with the maintenance of a simple, icon-like image of the face, whereas the left frontal activity represents a more elaborate face representation that is created after longer retention delays and is more easily maintained (Haxby et al. 1995). Regions in the frontal gyrus were found to be activated during visual imagery of faces but not during face perception (Ishai et al. 2002). During visual imagery, the frontal regions evoke top-down control for generating and maintaining visual images of faces. However, it is debated whether this process is category-selective and evokes different activation patterns in response to faces and objects (Mechelli et al. 2004) or not category-selective, as visual imagery of different objects evokes the same non-content related activity in the frontal cortex regardless of object category (Ishai et al. 2000). These studies provide evidence for the involvement of the prefrontal areas in cognitive control, working memory, and perception. This suggests that these regions may represent a connection between top-down cognitive control processes and bottom-up perception and hence these areas may also be involved in familiarity judgment by comparing the internally stored information about a person to the perception of a face (Heekeren et al. 2004; Baldauf and Desimone 2014). Neuropsychological evidence further support this hypothesis, as damage to the right prefrontal cortex causes false recognition, which is defined as the tendency to mistake unfamiliar faces for familiar ones without impairing other face-related processing (Rapcsak et al. 1996). False recognition in frontal patients is suggested to result from impaired strategic decision making and monitoring to determine whether a face is truly familiar, thus representing a control area between memory and perception.
